# The Making of a Modern Hospital

**Published:** 1911-10-14

**Authors:** 


### THE MAKING OF A MODERN HOSPITAL.

### II.—CLASSIFICATION OF MODERN HOSPITALS.

For purposes of classification, it is convenient to divide the institutions for the care and relief of the sick into two very distinct and definite classes. In the one are included all institutions which are destined to receive patients with a view to endeavouring to cure them of their ailments; this is the large class of hospitals or infirmaries. In the other we conveniently group institutions which cater for those patients who suffer from chronic or incurable diseases—cases, that is, in which the hope of an ultimate cure is not absolutely illusory, though the prospect of restoring the patient to health is a remote one; this is the equally large class of asylums. It is well to lay stress on the importance of differentiating between these two classes, for the public still appears to be in some doubt as to what constitute the proper functions of an asylum or of a hospital. The difference between the two classes is largely one of degree; it is not indeed absolute, but it is sufficiently important to warrant the institutions in the one class being built, managed, equipped, staffed, and utilised in a manner very different from that in which institutions in the other are built or managed. The two-class division has introduced two new words into our language, or, rather, we have taken these two words from the German which frequently employs them in the technical sense in which they will be used in these articles—namely, "hospitalisation" and "asylisation." By the former we mean the admitting into an institution of the first class of such patients as are afflicted with curable disease, or with a condition which is still amenable to medical treatment in the broadest sense. By the latter we imply the admission into institutions of the second class of patients whose condition is such that medical treatment holds out little chance of permanent relief. A hospital is not a home for the dying; it is not the place for a patient afflicted with general paralysis of the insane, for an incurably blind patient, or a permanently and incurably deformed person; all these cases need "asylisation" not "hospitalisation," and their proper place is in a specially built and equipped institution where they can be looked after by specially trained attendants. It may and does, of course, happen that an incurably diseased person suffers from a temporary and acute form of some intercurrent malady which necessitates prompt medical treatment; in such a case he may be legitimately transferred to a hospital, there to receive the treatment which he needs. Similarly it may happen that a patient in a hospital develops symptoms of a chronic and incurable disease which make his retention in an asylum necessary. But the fact remains that the differentiation between the two classes stands as a convenient generalisation which it is well to bear in mind. For when it is forgotten we get, as a result, the inclusion among hospital patients of persons who are obviously not in a state to benefit by medical treatment. To the lay mind it may seem callousness on the part of a hospital to reject from its wards a patient who is suffering from cancer in an advanced degree. Yet, from a hospital point of view such rejection is not only sound policy but it is a kindly policy as well. The admission of this one patient, whose condition may perhaps be assuaged but cannot be cured by treatment so far as we know at present, means the closing of one bed for a period of from one to six or more weeks; it means the exclusion of so many urgent or curable cases which might have been admitted into that one bed during that period. The proper place for the advanced cancer case is an asylum—a home for the incurable or dying, and it is mistaken kindness and a perverse "humanitarianism" that insist that such a patient should be admitted into a hospital. The latter institution is meant for the reception and treatment of patients who are likely to find benefit from medical science and skill. The best hospitals, in theory at least, are those which treat the largest number of patients successfully in the shortest possible time.

Having once grasped the difference between these two big classes of institutions, the reader is in a position to realise that we have nothing to do with the second class in this series of articles. We are not concerned, directly at least, with the modern asylum, but with the modern hospital, and the definition of a hospital that we would presume to give here is the following: "A hospital is an institution destined for the reception and treatment of patients whose condition it is possible to cure, or, if cure is impossible, so to relieve that they are capable of being sent home in an improved state of health."

Of hospitals in this sense of the term there are several varieties. Modern classification groups these into three divisions, the first of which is conveniently sub-divided into two classes as follows:

1. General hospitals: (*a*) large hospitals; (*b*) small or cottage hospitals.
2. Special hospitals.
3. Sanatoria.

Properly speaking there are only two classes, namely, general and special hospitals, for the sanatorium is really only a variety of the special hospital. For the sake of convenience, however, it is permissible to give the sanatoria a special division and to deal with them separately.

By a general hospital we mean an institution which receives and treats all classes of patients, no matter from what diseases they suffer, provided the general rule above enunciated, that a hospital should only receive patients who can benefit by the treatment, is held in view. Such a hospital is divided, roughly speaking, into two departments—a medical and a surgical side—but it has, in addition, a large number of special departments which are devoted to the treatment of special disorders. It is, in fact, a microcosm of organised medical relief to the sick, and to carry out its functions with success it should be equipped, managed, and constructed on the best lines in accordance with the most modern principles. The differences between a large and a small general hospital is merely one of size. It is convenient to group those institutions which possess more than a hundred beds under the head of large general hospitals, and to refer to those which have fewer beds as "small" or "cottage" hospitals. The same distinction with regard to size applies to the special hospitals, but it is rarely necessary to lay stress upon it since few special hospitals can compare with any of the medium-sized large general hospitals in this country and elsewhere.

By a special hospital, on the other hand, we mean an institution which only receives and treats patients suffering from special disorders. The number and variety of these institutions have increased enormously during the last twenty-five years, largely owing to the efforts of specialists to magnify the importance of the subjects in which they are engaged. Thus in London, to mention only one centre, we have special hospitals for diseases of the skin, for diseases of the eye, for children, for women, for venereal diseases, for tropical diseases, for diseases of the throat, for paralysis, for nervous diseases, for deformities, for urinary diseases, for fevers, for diseases of the chest, for hip disease, for cancer, for maternity cases, for consumption, for diseases of the teeth, for diseases of the rectum, and for stone, while in addition we have institutions established for some special object, which, although they are presumably general hospitals, are yet, owing to the fact that they limit their treatment in accordance with certain empirical doctrines, properly to be classified among special hospitals, such institutions being the Homœopathic and Temperance Hospitals, and the Anti-Vivisection Hospital at Battersea. A special hospital, then, is an institution established for a special object and dealing with a special class of patients. In the majority of cases its justification for existence depends largely on the good that it does and its usefulness to the community. Thus it is generally conceded that children's hospitals are desirable; similarly, special institutions for the treatment of diseases of the eye or ear, and of women may be considered as having proved their usefulness by the excellent work that they have done in the past and are doing to-day. But in general, special hospitals are unnecessary in cities which possess good, well-equipped, and well-staffed general hospitals provided with the requisite special departments for treating certain classes of disease. Their indiscriminate multiplication is therefore much to be deprecated since they tend to divide what may be called "hospital energy," and are uneconomical. To this rule one class of special hospital is probably an exception, this being the fever hospital, or, generally, the hospital for the treatment of infectious cases. It is quite true that every general hospital ought to have, and in the majority of cases does possess, an isolation department, but there are very serious objections to receiving infectious cases in a general hospital which is inhabited by "clean" cases. The admission of such infectious cases means the duplication of the staff and adequate isolation provision—in other words, the creation of a special hospital within the limits of the general hospital. No great advantage—beyond that of centralisation—is gained by this policy, and it is therefore a better plan to provide special fever hospitals at some distance away from the zone of the general hospitals for the reception of such cases. Formerly, when the risks of infection, owing to our imperfect knowledge of carriers and prophylaxis, were greater than they are to-day the necessity for special fever hospitals was even more apparent. Nowadays there is some justification for the hospital experts who object to the establishment of such institutions on the score that they tend to split energy and increase expenditure. These objectors maintain that every general hospital ought to be provided with fever wards and an isolation department, and hold that it is perfectly possible to ensure the safety of "clean" patients by adopting proper and easily observed precautions. This theory has obtained some popularity in Germany, where the newer hospitals have been provided with excellent and up-to-date fever departments—a policy which has been adopted after a full consideration of the merits attaching to the English system of specialisation. Nevertheless, there are good authorities in Germany who contend that it would be better to follow the English system and establish separate and independent fever hospitals, retaining only a small isolation ward for the reception of doubtful or definitely infectious cases in the general hospital.

When we come to the third class, the sanatoria, we see at once that these institutions, properly speaking, have no claim to being considered as distinct from the second class. They are special hospitals in so far as they serve for the reception and treatment of special cases. By far the larger number of them cater for consumptives or for patients afflicted with the drug habit, though it is customary to restrict the term sanatorium to institutions which receive and treat only consumptives. They are institutions which provide the connecting link between hospitals and asylums, for they approach on the one side very closely to the former, while on the other they trench on the field of activity of the latter. The class of patient they receive is technically chronic—that is to say, the period of time demanded for their cure is so great that to receive such cases in a general hospital would mean the "holding up" of a number of beds for an indefinite period to the exclusion, consequently, of so many urgent cases who might have occupied these beds. This fact appears to be a good justification for their existence, but already there is a tendency to deplore their enormous increase and to criticise unfavourably their results. Recent statistics tend to show that their importance as a means of cure has been overrated, and a growing school insists that they ought properly to be classed as asylums and to be established solely on what is known as the Swedish model. A discussion of this question and the many side issues involved is, however, beyond the scope of these articles.

